# Clinical and Molecular Properties of Human Immunodeficiency Virus-Related Diffuse Large B-Cell Lymphoma

**DOI:** 10.3389/fonc.2021.675353

**Published:** 2021-04-29

**Authors:** Pedro S. de Carvalho, Fabio E. Leal, Marcelo A. Soares

**Affiliations:** ^1^ Programa de Oncovirologia, Instituto Nacional do Câncer, Rio de Janeiro, Brazil; ^2^ Departamento de Genética, Universidade Federal do Rio de Janeiro, Rio de Janeiro, Brazil

**Keywords:** NHL, DLBCL, HIV, ABC, GCB, PLWH

## Abstract

Non-Hodgkin lymphoma is the most common malignancy affecting people living with HIV (PLWH). Among its several subtypes, diffuse large B-cell lymphoma (DLBCL) is an important manifestation within the HIV-infected compartment of the population. Since HIV is able to modulate B cells and promote lymphomagenesis through direct and indirect mechanisms, HIV-related DLBCL has specific characteristics. In this review, we address the clinical and molecular properties of DLBCL disease in the context of HIV infection, as well as the mechanisms by which HIV is able to modulate B lymphocytes and induce their transformation into lymphoma.

## Introduction

Historically, lymphomas have been assigned as either Hodgkin or non-Hodgkin types according to histological features [for example, the Reed-Sternberg cells (1, 2)], clinical presentation and response to therapies (3, 4). Non-Hodgkin lymphomas (NHLs) are a large spectrum of diseases that arise from lymphocytes at different stages of development, affecting virtually any organ (5). Also, NHLs are more frequent than Hodgkin lymphomas (6). According to the latest Globocan estimates, NHLs show incidence and mortality of 5.8 and 2.6 per 100.000 inhabitants worldwide, respectively (6). Currently, the World Health Organization (WHO) recognizes more than 60 distinct entities as members of the NHL group, excluding lymphoproliferative disorders and non-malignant manifestations (7). NHLs may arise from B lymphocytes, T lymphocytes or NK cells (7). Generally, NHLs subtypes from B cells are more common and represent about 85% of all cases (8, 9). Although data from Globocan represent collective incidence of NHLs, the distribution of subtypes is not homogenous and varies according to the study population (10). Among B cell-derived NHLs, the diffuse large B cell lymphoma (DLBCL) is consistently reported as the most common manifestation worldwide, followed by follicular lymphoma (FL, especially in Central and South Americas) and chronic lymphocytic leukemia (CLL, especially in southeast Europe) (10, 11). Individuals with Caucasian ancestry were associated with increased incidence of DLBCL, FL and CLL when compared with subjects with Hispanic, Asian or African ancestries (12). Few epidemiological data address specifically DLBCL and, in general, NHLs are reported as a group. However, a study performed with the US population from 1975 to 2017 showed DLBCL incidence estimates of 5.6 per 100.000 individuals per year (13). Also, between 45-60% of all NHL cases reported in Central and South America were DLBCL (14). A study performed with 27,796 NHLs diagnosed between 2000 and 2016 in Sweden reported DLBCL as the most common subtype, accounting for 35% of all cases (15). Those results indicate the relevance of DLBCL as a frequent NHL manifestation. The epidemiological data described above refers to the general population, however, it is known that specific subpopulations, such as people living with HIV (PLWH), harbor even greater burden of NHLs and DLBCL.

Among the risk factors linked with NHL development, immunosuppression is one of the well-documented. For example, solid organ transplant recipients had six-fold higher risk of developing NHL than the general population (16). In this scenario, HIV infection and lymphomagenesis were also extensively associated due to the immunosuppression induced by HIV infection (8). Indeed, before the advent of highly active antiretroviral therapy (HAART), HIV-infected patients had increased incidence of cancers associated with infection by other oncogenic viruses, such as Kaposi’s sarcoma (HHV8 - human herpes virus 8), cervical cancer (HPV - human papillomavirus) and NHL (EBV – Epstein-Barr virus) (17). Those cancers, including the group of NHLs, were subsequently named AIDS defining cancers (ADCs), given their relationship with HIV-associated immunosuppression, while all the other tumors observed among PLWH were called non-AIDS defining cancers (NADCs) (17, 18). Specifically, PLWH had a 113-fold higher risk of developing NHLs than uninfected counterparts (18). Accordingly, it was observed that the increase in NHL cases during the 1980s in USA was due to, in large part, the increase in AIDS-associated NHL (19). Nevertheless, after the advent of HAART, the epidemiology of cancers in PLWH suffered significant changes. With access to HAART, restoration of immunocompetence and aging of PLWH, the incidence of ADC started to decline while the incidence of NADC increased (20). According to a study performed with an European database, NHL incidence was 462.6 per 100.000 HIV-infected people that did not receive treatment, while the incidence was 205.1 per 100.000 HAART-treated patients (21). However it is clear that, despite the decreasing number of NHL cases in PLWH after the introduction of HAART, their estimate is not yet comparable to that of the general population and the incidence remains higher in HIV-infected people (20). Currently, even in the post-HAART era, NHLs are still reported as the most common neoplasia in PLWH (22). Among the numerous subtypes of NHLs, DLBCL and Burkitt lymphoma are the most frequent manifestations in PLWH (23, 24). In fact, about 60-70% of all NHLs from HIV-infected subjects are reported as DLBCL (24–26), indicating its relevance in this specific subcompartment of the population.

Regarding clinical presentation, NHLs may affect lymph nodes (nodal manifestation) or other tissues outside lymph nodes (extranodal manifestation), among which gastrointestinal tract and central nervous system are relatively common sites (27). NHL patients may present lymphadenomegalia and/or B symptoms (≥ 10% weight loss during the last six months, night sweating and fever ≥ 38°C) (28). NHL staging is based on the Ann Arbor system, which defines four stages according to the disease dissemination (29, 30), while the International Prognostic Index (IPI) is used for the prognosis of NHL patients (31).

In the current review, we address one of the most relevant subtypes of NHLs, DLBCL. We cover its molecular characteristics and its specific features when manifested in PLWH.

## The Molecular Basis of DLBCL

**Figure 1:**
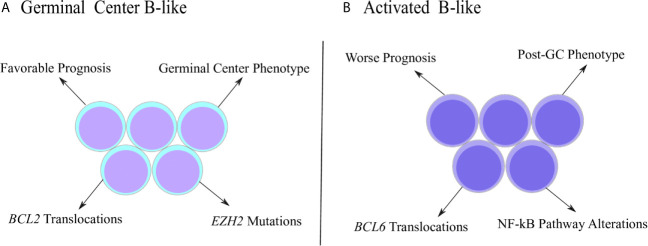
Distinctive characteristics of DLBCL subtypes. **(A)** Germinal center B-like (GCB) subtype was originally characterized by a gene expression pattern that resembled the germinal center (GC) phenotype. GCB cases are associated with better prognosis, as well as molecular properties, such as increased frequency of *BCL2* translocations and *EZH2* mutations. **(B)** Activated B-like (ABC) subtype was described with a gene expression pattern associated with the post-GC phenotype. ABC subjects show worse prognosis and specific molecular properties, such as higher frequency of *BCL6* translocations and alterations involving the NF-kB pathway.

The DLBCL is not a homogenous disease and can be further stratified into subtypes according to gene expression signatures (**Figure 1**). The first report that identified DLBCL subgroups based on molecular properties was performed by Alizadeh and coworkers (2000). Using microarray assays, the authors investigated the expression pattern of genes related with B cell development and lymphomagenesis, which allowed the clusterization of DLBCL samples into two main subgroups called GCB (germinal center B-like) and ABC (activated B-like) (32). The GCB subtype was characterized by elevated expression of genes associated with germinal center (GC) phenotype, such as BCL-6, BCL-7A, LMO2 and others, while the ABC subtype was characterized by low or undetectable levels of GC markers accompanied by high expression of genes related to plasmacytic differentiation, a post-GC phenotype (32, 33). Interestingly, even though the ABC subtype initiates the transition towards the pro-plasmacytic program, as observed by the high levels of IFR4 and other markers (34), ABC cells are incapable to conclude the plasmacytic differentiation because they fail to stimulate transcription factors ultimately required for the acquisition of plasma cell phenotype (35), such as Blimp-1 (36, 37). In fact, Blimp-1 mutations that inhibit its expression or destabilize the protein were described exclusively in ABC cases (38), emphasizing its incapacity to follow the plasmacytic program completely. Therefore, ABC subtype represents an abnormal stage of B cell ontogenesis in which the differentiation is arrested in between the end of germinal center reaction and the commitment with plasma cell generation (33). The distinction between GCB and ABC is relevant since it adds prognostic value and, classically, ABC patients exhibit worse survival estimates than GCB subjects (32).

After the first description of DLBCL subtypes, new methodologies for subtype identification were published. Wright and coworkers (2003) identified a list of 27 genes with predictive value to distinguish between ABC, GCB and a third group called unclassified, since it did not fit into either of the two categories established (39). In agreement with the original report (32), the subtypes described according the list of 27 genes also had prognostic value and the overall survival estimates were 31%, 59% and 47% for ABC, GCB and unclassified cases respectively (39). On the other hand, the Hans algorithm proposes subtype identification based on immunohistochemical assessment of three key proteins: BCL-6, IRF4 and CD10. According to this method, the authors were able to distinguish between GCB (CD10+, IRF4- and/or BCL6+) with overall survival of 76% and non-GCB cases (CD10- and BCL6- or CD10-, BCL6+ and IFR4+) that showed only 34% overall survival estimates (40). Another technology applied in DLBCL subtype identification is the Lymph2Cx panel, which interrogates the expression levels of 20 key genes using digital gene expression analysis (34). It is a method especially relevant for formalin-fixed paraffin-embedded (FFPE) samples and also aggregates prognostic values like the abovementioned methodologies (34). Interestingly, even though DLBCL subtype identification had its prognostic relevance confirmed in several reports, its predictive value was not observed among HIV-infected patients with the disease (41), indicating that the lymphoma has unique properties in the HIV infection context that must be addressed apart.

Molecular events that happen during B lymphocyte development are also a source of genetic alterations found in DLBCL (42). Besides somatic hypermutation and class switch recombination (43), nucleotide substitutions catalyzed by AID (activation-induced deaminase) may also produce somatic mutations and large-scale alterations, such as chromosomal translocations (33). Break sites in genes commonly translocated in DLBCL are often AID-targeted regions (44–46). Moreover, DLBCL samples exhibited proto-oncogene mutations at sites recognized by AID (47). Therefore, deregulation and errors resulting from B lymphocyte ontogenesis may also promote DLBCL development.

Chromosomal translocations consists on the exchange of DNA segments between distinct chromosomes and are among the most common DLBCL genetic alterations (48). The pattern generally observed is the transfer of a protoncogene segment to a site downstream of an immunoglobulin locus, usually immunoglobulin heavy-chain (IgH) (49). This mechanism positions protoncogenes under the regulatory control of IgH loci and not their original regulatory regions, affecting gene expression (33, 46, 49). BCL2, BCL6 and MYC are common translocation targets in DLBCL (50). BCL2 translocation is described as t(14;18) because it is the transfer of a chromosome 18 segment containing BLC2 to an IgH downstream site on chromosome 14 (51). This translocation is not observed in ABC subtypes, but is especially frequent among GCB cases, affecting 35-40% of cases (52, 53). Also, evidence suggests that t(14;18) is acquired early in ontogenesis during VDJ recombination breaks (54). BCL2 translocation was associated with increased protein expression, whose antiapoptotic effect favors the survival of transformed cells (52, 53, 55). In ABC subtype, increased expression of BCL2 was also reported, but was associated with chromosomal duplications and not t(14;18) (55). Moving forward, BCL6 translocations were described either affecting IgH locus on chromosome 14 or non-Ig loci. The t(3;14) event occurs between IgH and a fragment of chromosome 3 containing BCL6 (56). Even though this translocation affects only 10% of GCB cases, 25% of ABC samples comprise that event (57). This may also be another mechanism by which the ABC subtype is unable to completely assume the plasmacytic program, since t(3;14) positions BCL-6 under the command of IgH regulatory regions and prevents its complete inhibition after the end of the germinal center reaction (58). ABC samples carrying t(3;14) expressed higher levels of BCL6 than non-carriers of the same subtype (57). However, in analysis without subtype stratification, BCL6 expression was not significantly affected by t(3;14) (57, 59). On the other hand, BCL6 translocation with non-Ig partners (such as histone H4) were associated with increased gene expression (60).

MYC translocation towards the IgH locus was reported as t(8;14) (61). It is a less common event than BCL2 and BCL6 translocations, affecting about 10% of DLBCL cases (62, 63). Among t(8;14) carriers, the majority belongs to GCB subtype (64). MYC is a transcription factor related with cell cycle and survival, and therefore its increased expression after juxtaposition to IgH locus also contributes to carcinogenesis (65). BCL2, BCL6 and MYC translocations are not mutually exclusive and may occur together in the same patient, forming the double-hit and even triple-hit lymphomas (66, 67). A study with 155 DLBCL subjects showed that 2.3% were double-hit for MYC/BCL2, 2.3% were double-hit for MYC/BCL6 and 0.8% (one patient) was triple-hit and carried the three translocations (68). In general, carriers of multiple hits (two or three translocations) are associated with worse survival (55, 64, 69).

Besides chromosomal translocations, some point mutations in DLBCL were also reported. Nucleotide substitutions, deletions and duplications were described in several genes, such as BCL6, MYC, PAX5, PIM1, RhoH and others (47). A large genomic analysis performed with 1,001 DLBCL cases described a list with the 60 most frequently mutated genes (70). Among them, they described mutations in MYC, PAX5, BCL2, CARD11, CDKN2A and other targets, indicating the genetic heterogeneity found in this cancer. However, the authors also observed some common patterns. For example, BCL2, with known antiapoptotic properties (71) and CARD11, a gene associated with lymphocyte activation (72) suffered missense mutations or copy number gains, while genes involved with cell cycle inhibition, such as CDKN2A (73) suffered nonsense mutations or copy number losses (70). Overall, mutations found in DLBCL favored pathways associated with survival or cell cycle progression and blocked pathways with the opposite effect (74). Accordingly, CARD11 was pointed as an important oncogene for DLBCL development in other reports (75, 76).

The distribution of some mutations also varies according to the DLBCL subtypes (77). Specifically, in GCB cases lesions that allow the persistence of the germinal center transcriptional program are observed, preventing the transformed cells to move forward to the following stages of normal B cell development, a process called “locking in” (33). One of the main components responsible for keeping the germinal center phenotype in GCB cases is the EZH2 enzyme, a known repressor of transcription factors needed for plasma cell differentiation, such as Blimp1 and IRF4 (78). The inhibition occurs with the triple methylation of lysine 27 residue on histone H3 located in regulatory regions of those genes (78). Thereby, the enzyme suppresses the pro-plasmacytic program and confines the transformed cell within a GC phenotype (58). Gain of function mutations in EZH2 are found in 20% of GCB cases and are not found in ABC subtype (79), a pattern already described in the literature (70, 74, 80). Those are missense mutations in which a tyrosine residue from the catalytic site of the enzyme is replaced by another amino acid, affecting directly its affinity for the substrate, which results in hypermethylation of the target regions and leads to an inhibition even more pronounced than by wild-type EZH2 (81). On the other hand, ABC subtype is characterized by alterations in genes of the NF-kB pathway (70, 80). NF-kB is a transcription factor important for B cell development, mediating proliferation, survival and apoptotic pathways (82). The NF-kB pathway is constitutively activated in ABC subtype (83). In vitro administration of NF-kB pharmacological inhibitors was toxic exclusively in ABC cell lines and did not have an effect in GCB models (83). Some genetic lesions are responsible for NF-kB constitutive activation. TNFAIP3, a NF-kB repressor, suffers deletions or loss of function mutations in 30% of ABC cases (84). In parallel, MYD88, an activator of NF-kB, showed gain of function mutations in 29% of ABC samples (85). Alterations in those genes are rare or not reported for GCB (70, 84, 85).

Regarding DLBCL treatment, the main option is chemotherapy. Initially, a combination of drugs called CHOP (cyclophosphamide, hydroxydaunorubicin, vincristine/oncovin and prednisolone) was used (86–88). The advent of an anti-CD20 monoclonal antibody (rituximab) with antitumoral activity (89) led to its addition to chemotherapeutic regimens, creating the R-CHOP treatment. R-CHOP-treated patients showed significantly greater survival estimates when compared to CHOP-treated subjects (90–93). For example, progression-free survival estimates were 66% and 45% for R-CHOP and CHOP groups, respectively (90). Currently, R-CHOP is the principal treatment option for DLBCL, but 30% of relapsed cases are still observed (90). More recent studies are searching for treatment strategies according to DLBCL subtypes, especially for ABC given its worse survival performance. Since NF-kB pathway function is crucial in ABC subjects, a study raised the hypothesis that bortezomib, an NF-kB inhibitor, could exert selective efficacy on this subtype (86). However, the combination of bortezomib and R-CHOP did not improve survival estimates when compared to R-CHOP regimen alone (94, 95), indicating that new subtype-specific targets must be studied. Accordingly, the combination of R-CHOP and ibrutinib, a BTK inhibitor, resulted in better survival estimates in ABC patients that were younger than 60 years when compared to R-CHOP alone (96). Although statistical significance was not observed, the R-CHOP and lenalidomide combination showed a trend towards an improved survival among ABC patients with worse IPI scores (≥ 3) (97).

## HIV-Induced Modifications in B Cells

**Figure 2:**
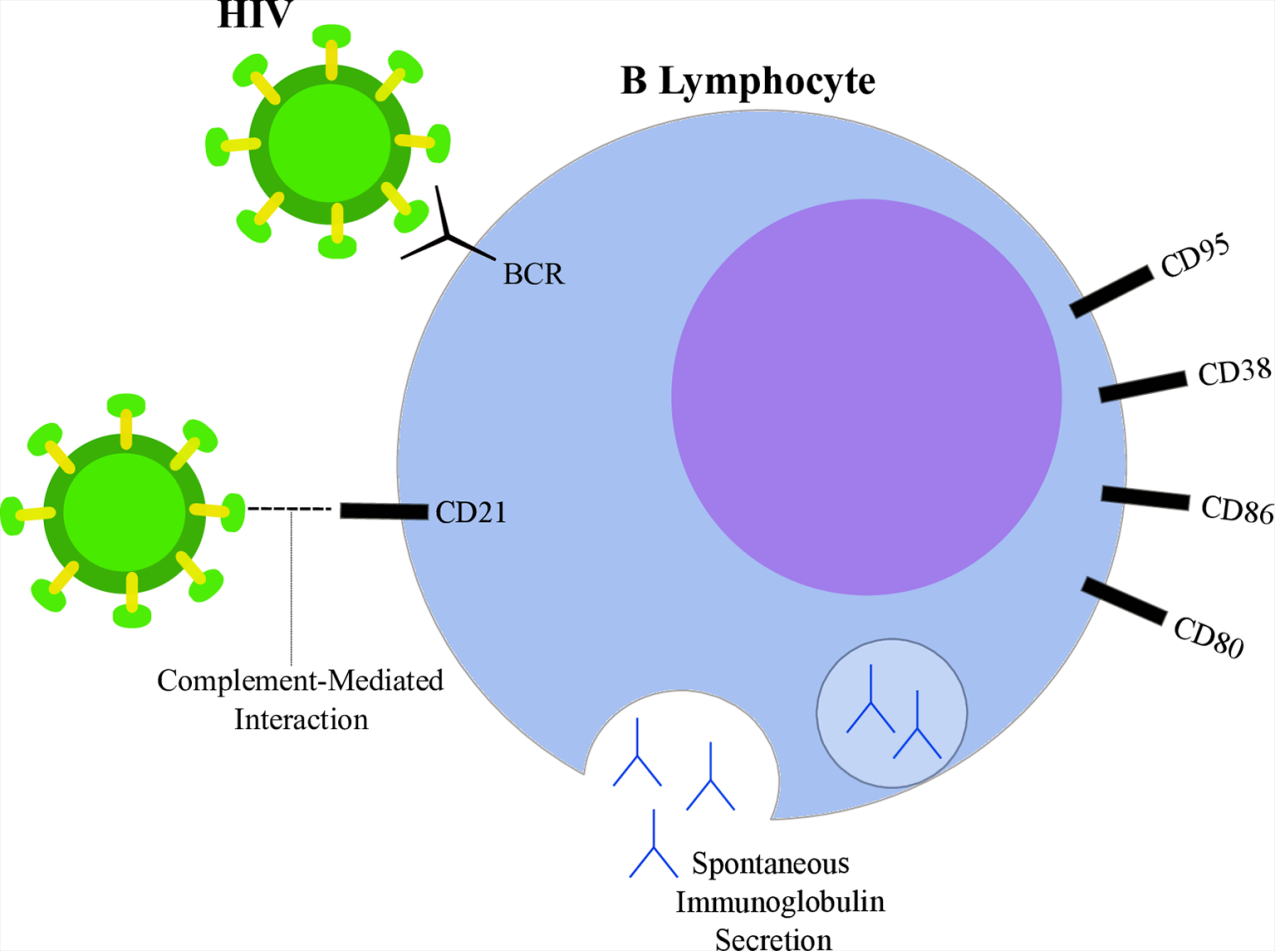
Highlights of HIV-induced B lymphocyte alterations. HIV particle (green) is able to bind B lymphocyte (blue) through direct interactions with surface immunoglobulins (B-cell receptor, BCR) and through CD21 interactions mediated by complement proteins. Spontaneous immunoglobulin secretion leading to hyperglobulinemia is observed in the context of HIV infection, as well as alterations in surface markers, such as increased expression of CD80, CD86, CD38 and CD95.

Although the general molecular mechanisms involved with DLBCL pathogenesis are well documented and summarized above, the specificities of DLBCL in an HIV-infected context are yet unclear. Despite T-CD4 lymphocyte depletion being one of the hallmarks of HIV infection, viral-induced effects are also reported for the B cell compartment (**Figure 2**). Knowing that B lymphocytes are the progenitor cells of DLBCL, we will first approach how HIV is able to exert its influence over B lymphocytes either by direct or indirect mechanisms throughout its life cycle in this section. As suggested by Moir & Fauci, most of the B cell alterations are due to unbalanced distribution of subpopulations, that is, while certain subgroups of B cells suffer a shrinkage under the influence of HIV, others will undergo enrichment (98) and even participate in lymphomagenesis.

Studies suggest that HIV is able to directly interact with surface molecules on B lymphocytes. Despite not expressing CD4, interaction between the viral glycoprotein gp120 and membrane immunoglobulins from the variable heavy chain 3 (VH3) family has been reported (99). Moreover, gp120 was shown to interact with C-type lectin receptors on B lymphocytes (100, 101). CD21 is another known binding site of HIV in B cells through interactions mediated by complement proteins (102). However, whether this interaction is able to induce direct effects on lymphocytes is still being discussed (98). On the other hand, a known participation of CD21-dependent interactions contributes to the maintenance of a extracellular reservoir of surface-bound viral particles potentially transmittable to T-CD4 cells (103, 104). Furthermore, HIV may also influence B cells through indirect mechanisms by altering the cytokine secretion pattern of different cell types. Indeed, several cytokines were found overexpressed in plasma samples from PLWH when compared to uninfected controls (105). For example, HIV increased T-CD4 lymphocyte spontaneous IL6 secretion (106), IL10 and TNFα production by monocytes exposed to viral glycoproteins (107) and IFNα secretion by plasmacytoid dendritic cells (108). Those cytokines contribute to B cell activation, differentiation (109, 110) and may also guide the modifications observed upon HIV infection.

A common feature observed within the B cell compartment during HIV infection is the acquisition of a hyperactivation phenotype, characterized by a plethora of physiological alterations. One of the first studies to suggest chronic B cell activation in PLWH was performed in 1983 by Lane and coworkers. The authors indicate that, although the peripheral B cells extracted from AIDS donors were less responsive to antigenic stimulation, those cells exhibited spontaneous secretion of IgG, IgA and IgM at levels 10 times higher than HIV-uninfected controls (111). In agreement, the increased serum levels of immunoglobulin molecules, a state defined as hyperglobulinemia, was also associated with HIV infection (112–114). Analyses regarding the cellular source of immunoglobulin secretion were suggestive of a polyclonal B cell activation in PLWH (115). Taken together, these data suggest a shift in the B cell compartment towards a pro-plasmacytic pathway, since increased antibody secretion was reported under the influence of HIV infection (98, 116). This pattern was also observed when the peripheral B cells from PLWH were shown to be enriched in a population with low levels of CD21. This subgroup (CD21low) was associated with diminished proliferative capacity after antigenic stimuli and with plasmacytoid morphological features (116). Indeed, both CD21 downregulation and decreased proliferative response are related to cell priming into the plasmacytic differentiation program (117). The favoring of plasmacytoid pathways upon HIV infection was reported by others (118, 119), emphasizing the viral effect on reorganizing the pool of B cells towards a less responsive and terminally differentiated route. Interestingly, while CD21low cells showed enhanced antibody secretion in PLWH (116), they were not able to spontaneously secrete immunoglobulin in uninfected individuals (117). Another aspect of HIV-induced B cell hyperactivation is the modification of surface markers, such as the increased expression of activation-related molecules (120). Nevertheless, although the baseline levels of CD86 and CD80 were higher among HIV subjects, their B cells were not capable of upregulating the expression of those markers upon receiving proper stimulation (121). Similarly, CD38 levels were higher in B cells from HIV-infected donors, but those cells were also more susceptible to apoptosis since the levels of the proapoptotic CD95 molecule were increased (122). Those results are in agreement with, and help to delineate the paradoxical nature of the hyperactivation phenotype: while B cells show evidence of activation at resting state, they are poorly responsive to adequate stimuli, which contributes to impaired humoral responses.

Additional HIV-induced effects on the B cell compartment were reported in the literature, such as effects on memory B cells and immune exhaustion, a state defined by diminished proliferative response and effector functions upon antigenic stimulation (123). As described above, the loss of CD21 expression is a valuable marker of HIV disease in B cells. The CD21low subpopulation forms a heterogeneous cluster that reveals multiple B cell modifications under influence of HIV (124), including evidence of immune exhaustion. Although CD21low cells were initially associated with plasmacytoid features, confirming the hyperactivated phenotype, not all of them belong to the pro-plasmacytoid compartment (98). A fraction of CD21low cells characterized by low levels of CD27 and high expression of FCLR4 has been observed in HIV+ patients (125). The expression of those markers also described a specific subpopulation of memory B cells found originally in tonsils (126). Therefore, those CD21LOW CD27- FCRL4+ cells were subsequently named as tissue-like memory cells and were shown to be enriched in PLWH (127). Interestingly, evidence of premature exhaustion was found in tissue-like memory cells, demonstrating their poor responsiveness to appropriate BCR stimulation when compared to conventional memory B cells (127, 128). Those results indicate the expansion of an abnormal B cell subpopulation in PLWH, suggesting the enrichment of an exhausted memory B cell phenotype and impaired humoral responses.

In this scenario, an interesting topic is whether highly active antiretroviral treatment (HAART) is able to reverse HIV-induced B cell modifications. Although it is well established that the therapy is capable of minimizing B lymphocyte alterations, the extension of that activity is suggested to be partial and not valid for all the observed HIV-induced effects. Hyperactivation and hyperglobulinemia are examples of alterations completely resolved by HAART (129). When compared to untreated patients, immunoglobulin serum levels were significantly decreased after HAART administration and correlated with HIV viral load reduction (129, 130). However, effects regarding the reorganization of B cell compartments are incompletely reversed by HAART (131). As demonstrated by Moir and collaborators, some B cell subpopulations from HAART-treated PLWH reached percentages comparable to those found in uninfected donors, however the increase in resting memory cells was slow and incomplete, revealing the persistence of memory B cell deficiency (131). The partial restoration of that compartment was confirmed by others, indicating that HAART-treated subjects still have lower levels of memory B cells than uninfected counterparts (132). In agreement with those findings, the CD21low subset enrichment, one of the main B cell alterations upon HIV acquisition, was not completely abolished after HAART introduction, and CD21low counts were still significantly higher in HAART-treated patients than in uninfected controls, demonstrating a partial restoration of B cell subsets (133). In contrast, a report by Tanko and coworkers showed that, after HAART treatment of HIV subjects, the percentages of immature transitional, naive and memory B cell groups were equivalent to non-HIV individuals, except for plasmablasts, which continued to be enriched in PLWH even after HAART (134). Altogether, these results suggest that even after HAART, residual modifications in B cell subpopulations persist in PLWH.

## HIV-Induced Lymphomagenesis

Classically, HIV has been associated with lymphomagenesis (and carcinogenesis in general) because of its immunosuppressive activities. PLWH, while immunosuppressed, loose immunological vigilance over other pathogens and become permissive to oncogenic viral infection (135). Regarding lymphoma development, EBV (Epstein-Barr Virus) co-infection has a meaningful role (136, 137). EBV is a gamma-herpes virus able to infect B lymphocytes through interactions with CD21 (138). EBV infection is worldwide disseminated and progresses as an asymptomatic condition throughout life in the majority of carriers (139, 140). The activity of T lymphocytes is crucial to control EBV infection, assuring its asymptomatic status (141). However, HIV-induced immunosuppression favors EBV oncogenic activities, worsening the risk of lymphoproliferative diseases and lymphomas (142). The exact mechanisms by which EBV is able to induce oncogenesis are beyond the scope of this review and are addressed elsewhere (143–145). Some examples of oncogenic activities are found in EBV proteins produced during its life cycle. LMP1 (Latent Membrane Protein 1) is able to transform B cells and to stimulate lymphomagenesis (146). Its mechanism of action consists on the mimicry of CD40 physiological signaling, stimulating lymphocyte proliferation (147) and antiapoptotic pathways in an NFKB- (148) and Akt- (149) dependent manner. Another example is EBNA2 (EBV-encoded Nuclear Antigen 2), which also exhibits pro-survival activity and favors B cell lymphoma development (150). On the scope of reduced immunological surveillance, the HIV-infected context, especially in intravenous drug users, is associated with higher rates of HCV infection (151). HCV positivity was associated with lymphomagenesis (152). NHL patients with HIV showed even greater frequency of HCV infection than PLWH that did not exhibit cancer (153). HCV infection was also associated with worse overall survival and increased the risk of NHL development (153), suggesting another tumorigenic mechanism in HIV-infected subjects. Even though immunosuppression is one of the main oncogenic mechanisms of HIV infection, direct effects induced by the virus are also being reported as important contributors to lymphomagenesis.

As discussed in the previous section, HIV exerts influence over the host’s B cell population. In this scenario, effects on B lymphocytes contributing to lymphomagenesis have been reported. The B cell hyperactivation under HIV influence (111, 116, 133) is also associated with immunoglobulin (Ig) class switching (154, 155), one of the main sources of genetic variability in B cell lymphomas (33, 44, 46). Therefore, it may contribute to the acquisition of genetic lesions related to lymphomagenesis, such as chromosomal translocations (45, 156). Activation-induced deaminase (AID) is an enzyme whose activity is linked with the double-strand breaks needed for translocation events (43, 45). The interaction between the HIV gp120 glycoprotein and C-type lectin receptors on B lymphocytes is able to upregulate AID expression and, consequently, to trigger class switching events (100, 101). An additional triggering mechanism was observed where HIV particles with envelopes carrying CD40L host molecules stimulate B lymphocytes to, similarly, upregulate AID and proceed through Ig class switch (157). Indeed, previous reports had already observed high expression of immunological markers associated with Ig class switch in PLWH. Those studies showed that, when compared to HIV subjects that did not progress to lymphoma, high serum levels of IL6, IL10 and IgE were observed specifically among the ones that developed cancer until three years after enrolment (158, 159), reassuring the relevance of this pathway for HIV-associated lymphomas. Indeed, both IL6 and IL10 are cytokines associated with Ig class switch induction (109). Therefore, such favouring of AID expression and Ig class switch upon HIV infection may contribute to the acquisition of genetic lesions potentially able to drive lymphoma.

An additional mechanism recently proposed for HIV-induced carcinogenesis is the release of pro-tumoral exosomes. Exosomes derived from infected cells were enriched in HIV transactivator response element (TAR) RNA and associated with antiapoptotic properties (160). Chen and coworkers showed that exosomes from T cells or from PLWH plasma samples were able to directly induce cancer cell proliferation and oncogene expression through the EGFR/TLR3 axis followed by ERK1/2 phosphorylation (161). Interestingly, those effects were not observed when B lymphoma cell lines were used, apparently due to the lack of EGFR expression (161). However, exosome-derived microRNAs (miRNAs) were proposed as relevant biomarkers for Hodgkin lymphoma diagnosis in HIV-infected donors (162) and, therefore, it remains to be elucidated whether or not exosomes may have a role in lymphomagenesis or if their effect is cancer-specific.

Besides the contribution of HIV infection to class switching events, more recent reports have demonstrated direct participation of viral proteins during lymphoma development (163). For example, viral p17 matrix variants were able to stimulate proliferative and antiapoptotic pathways in B lymphocytes, facilitating their clonal expansion (164). The p17 variants with proliferative effect pass through a conformational change and expose an amino acid sequence originally enclosured in wild-type p17 three-dimensional structure (165). Even though the identity of the receptors involved in p17 signaling is not fully understood, interactions between p17 and CXCR1 or CXCR2 were demonstrated and associated with angiogenesis (166), another pro-tumoral effect. Besides p17, oncogenic activities were also reported for other viral proteins. Kundu and coworkers showed that Tat (transactivator of transcription) expression in mice favored B cell lymphoma generation in approximately 30% of animals (167). Accordingly, a study pointed that Tat was able to activate DNA repair proteins among B cells cultivated together with HIV (168), as well as to induce angiogenesis (169). More evidence indicates that Vpr (viral protein R) and Vpu (viral protein U) also participate in HIV-induced lymphomagenesis. For example, Vpr induced DNA double-strand breaks in infected cells (170), while Vpu was important for lymphoma adhesion in endothelial cells (171). Altogether, given the relevance of HIV in inducing specific lymphoma promoting pathways, we will approach the characteristics of HIV-related DLBCL in the next sections.

## Molecular Characteristics of HIV-Related DLBCL

The HIV-related DLBCL has molecular properties underpinning its specific clinical features described above. In this context, a report sought to investigate whether well-established prognostic genes would be equally informative for the HIV-related DLBCL. The authors reported that, although relevant for immunocompetent hosts, the expression of BLC2, Blimp-1 or FOXP1 did not correlate with patient outcome in PLWH. Interestingly, neither DLBCL subtype (ABC and GCB) was informative of survival in the HIV+ cohort (172), a pattern later confirmed by others (41, 173), suggesting that HIV-associated DLBCL is a particular disease and has its own molecular and pathological properties. In this section, we will address molecular differences reported in the literature when comparing HIV-DLBCL and IC (Immunocompetent)-DLBCL such as gene expression, miRNA levels and chromosomal organization, since the predictors applicable to DLBCL in the general population are not completely valid for PLWH (**Table 1**).

**Table 1:** Highlights of reports that explored the molecular characteristics of HIV-DLBCL.

Study	Population	Methodology	Main Findings
Teitell *et al.* (174)	U.S.A.	cDNA Subtraction	- Overexpression of *TCL1* among AIDS-related DLBCL when compared to non-AIDS lymphomas.
Nenasheva *et al.* (175)	Germany	cDNA Subtraction	- Upregulation of *a-myb* and *pub* observed in two biological samples from AIDS-related DLBCL when compared to normal B lymphocytes.- Upregulation of *a-myb* and *pub* in three biological samples of simian immunodeficiency virus (SIV)-associated monkey lymphomas.
Fedoriw et al. (176)	Malawi	WTS and IHC	- 2,523 genes found differentially expressed between HIV-DLBCL and IC-DLBCL applying an adjusted p -value of <0.1.- HIV status and DLBCL subtypes were not associated with OS differences.- IFNγ and IFNα were markers of positive prognostic among HIV-DLBCL only.- Ki-67 staining ≥80% was associated with lower survival among HIV-DLBCL.- cMYC/BCL2 co-expression was associated with lower survival independent of HIV status.
Maguire et al. (177)	HIV-DLBCL samples from ACSR and IC-DLBCL institutional cases	Digital Gene Expression Analysis, IHC and CNV Analysis	- Both HIV-DLBCL and IC-DLBCL groups were enriched in GCB subtype.- Increased frequency of Ki-67 >80% in HIV-DLBCL.- Reduced frequency of BCL2 positivity in HIV-DLBCL.- IC-DLBCL samples showed more copy number variations (CNVs) than HIV-DLBCL subjects.- 126 genes were differentially expressed between IC-DLBCL and HIV-DLBCL.- Gene set enrichment analysis indicated enhancement of genes associated with cell cycle progression, DNA replication and DNA repair in HIV-DLBCL.
Cassim et al. (178)	South Africa	IHC	- Higher frequency of Ki-67 >75% in HIV-DLBCL.- HIV-DLBCL patients with CD4 < 150 cells/mm³ had significantly worse survival than the HIV-uninfected counterpart.
Chao et al. (179)	U.S.A.	IHC	- Increased expression of cMYC, p27, BCL6, PKC-β2, MUM1, and CD44 among HIV-DLBCL subjects.- c-MYC expression was associated with worse 2-year mortality estimates in HIV-DLBCL.
Barreto et al. (180)	Brazil	IHC	- HIV-DLBCL samples exhibited 84%, 55%, 45% and 41% of positivity for CD20, CD10, Bcl-6 and MUM-1, respectively.
Madan et al. (181)	U.S.A.	IHC	- AIDS-DLBCL samples formed an intermediary cluster between GCB and ABC subtypes from non-AIDS subjects.
Thapa et al. (182)	Samples from ACSR	Microarray and qPCR	- Overexpression of miR-17, miR-106a, miR-106b, miR-18a, and miR-19a in AIDS-NHL (including DLBCL) patients when compared to normal B lymphocytes.- The activity of miR-106a and miR-106b significantly blocked the cell cycle inhibitor p21 using *in vitro* models.- miR-106a and miR-106b also exhibited positive effect on cellular proliferation.- Protein and mRNA levels of p21 were low or undetectable in AIDS-DLBCL and AIDS-BL (Burkitt Lymphoma) samples.
Phillips et al. (183)	South Africa	qPCR	- Higher expression levels of miR-21 among HIV-DLBCL when compared to IC-DLBCL.- High levels of miR-21 in HIV-DLBCL patients were associated with worse prognosis.
Thapa et al. (184)	Samples from MACS repository	qPCR	- miR-21, miR-122 and miR-222 were upregulated and miR-223 was downregulated in the serum of HIV-DLBCL patients when compared to HIV-uninfected tumor-free subjects.- miR-222 serum levels were higher among the HIV+ patients who went on to develop lymphomas (including DLBCL) when compared to HIV+ who did not progress to cancer.
Capello et al. (185)	Institutional cases	SNP-based microarray comparative genomic hybdridization, qPCR and methylation analysis	- HIV-BL exhibited lower copy number (CN) alterations than HIV-DLBCL cases.- The overall genomic complexity was similar between IC-DLBCL and HIV-DLBCL, however the distribution of genomic alterations was significantly different.- HIV-DLBCL showed more frequently 3p14.3 deletion (containing *FHIT* and the fragile site FRA3B) and 12q21.31 gains.- IC-DLBCL exhibited more often 18q gains (containing *BCL2*, *NFATC1* and others).- The tumor suppressor genes *FHIT* and *WWOX* were downregulated among HIV-NHL samples that carried either gene deletion or abnormal methylation patterns.
Morton et al. (186)	U.S.A.	IHC and FISH	- Only 31% of HIV-DLBCL subjects exhibited at least one of the three translocations assessed (*MYC/IgH*, *IgH/BCL2* and *BCL6/IgH)*.- *MYC/IgH* was the most common translocation among
			HIV-DLBCL cases.- Although subtype differences were not observed in HIV-DLBCL, in IC-DLBCL, *MYC/IgH* translocations were associated with GCB and *BCL6/IgH* were associated with ABC.
Deffenbacher et al. (187)	Samples from NCI AIDS and CancerSpecimen Repository and University of Nebraska	Microarray comparative genomic hybdridization	- Gene set enrichment analysis revealed enhanced representation of MYC, FAS and mTOR pathways in HIV-DLBCL when compared to IC-DLBCL.- HIV-ABC showed enrichment of MYC and ARF pathways when compared to IC-ABC.- The authors identified 13 recurrent copy number losses and 16 recurrent copy number gains among B-cell derived AIDS-related lymphomas.
Capello et al. (188)	Institutional samples from Caucasian HIV-infected patients	Sanger Sequencing	- HIV-DLBCL cases showed enrichment of *IGVH4* family and underrepresentation of *IGHV3* family when compared to normal B lymphocytes.- The same pattern was not observed among HIV-BL cases.
Yawetz *et al.*, (158)	Samples from the UCLA- MACS	Enzyme Imunoassay	- Higher serum levels of sCD23 and IgE were observed among HIV-infected subjects that went on to develop HIV-NHLs when compared to either HIV-uninfected or HIV-infected patients who did not progress to cancer.
Widney et al. (189)	Samples from UCLA-MACS	ELISA	- Increased serum levels of sCD27 in HIV-infected patients that developed HIV-NHLs when compared to HIV-infected patients who did not develop lymphoma.

ACSR. AIDS & Cancer Specimen Repository; MACS: Multicenter AIDS Cohort Study; UCLA-MACS, UCLA Multicenter AIDS Cohort Study; NCI, National Cancer Institute; WTS, whole transcriptome sequencing; IHC, immunohistochemistry; CNV, copy number variation; qPCR, real-time quantitative PCR; SNP, single nucleotide polymorphism; FISH, fluorescence in situ hybridization; ELISA, enzyme-linked immunosorbent assay.

One of the first reports to address gene expression signatures in HIV-DLBCL was performed by Teitell and coworkers (1999). Using cDNA subtraction techniques, they described overexpression of TCL1 (T-cell leukemia 1) oncogene in AIDS-related DLBCL samples when compared to non-AIDS tumors (174), which was later confirmed by the same group (190). Moreover, upregulation of a-myb and pub genes was also described in HIV-DLBCL cases, despite the limited number of human biological samples (n = 2) available in the study (175). More recent data compared the transcriptional profiles between 22 HIV-DLBCL and 14 IC-DLBCL from Malawi (Africa) using the whole transcriptome sequencing technology (176). The analysis revealed HIV status as a major contributor to differences observed in the expression levels of 2,523 genes. In fact, HIV-DLBCL samples were enriched in pathways related to hypoxia and cell metabolism when compared to the immunocompetent counterparts. Likewise, higher expression of IFNγ and IFNα were associated with better outcomes only in HIV-DLBCL patients (176). Another study found 126 differentially expressed genes when comparing the expression profiles between GCB subtypes from either PLWH or HIV-uninfected donors. The HIV-related GCB showed upregulation in genes associated with cell cycle progression, downregulation of cell cycle inhibitors and enhanced expression of DNA repair genes (177), indicating greater proliferative capacity. In fact, Ki67 staining, a proliferation marker, was reported as a valuable prognostic marker in HIV-DLBCL (172, 176). Consistently, stronger Ki67 staining was observed in HIV-DLBCL samples when compared to immunocompetent donors (177, 178). Immunohistochemistry studies have also provided valuable insights about HIV-DLBCL. The protein levels of cMYC, BCL6, PKC-β2, MUM1 and CD44 were significantly increased in HIV-DLBCL patients, while p27 levels were reduced (179). Among all those differentially expressed markers, cMYC positivity was associated with inferior survival in HIV-infected subjects (179), indicating new possible predictors for this population. cMYC and BCL2 simultaneous overexpression (double expressor lymphomas) comprehends a relevant inferior prognostic predictor in non-infected donors (191, 192). Some studies showed that the frequency of double expressors is similar between HIV-DLBCL and immunocompetent patients (193). However, its prognostic relevance was not yet addressed in the HIV-infected context, even though cMYC (179) and BCL2 (194) expression were described separately as prognostic factors for HIV-DLBCL. Similarly, another immunohistochemistry study detected high frequency (around 40%) of HIV-DLBCL samples positive for BCL6 and MUM1 (180).

Since DLBCL molecular subtypes are also determined by gene expression profiling (32), some reports investigated which one of them (ABC or GCB) were predominant in HIV-DLBCL. Those investigations, however, resulted in conflicting data. Some reports consistently show enrichment of GCB phenotype in HIV-DLBCL samples (172, 176, 177, 179, 180). On the other hand, enhanced expression of ABC markers was also demonstrated for those cancers (186, 187, 195). A third approach has indicated that HIV-DLBCL possess intermediate features between ABC and GCB, co-expressing markers of both subtypes simultaneously (181, 195, 196), which may explain discrepancies between reports. Indeed, a clusterization analysis performed by Madan and collaborators revealed that AIDS-related DLBCL samples formed an intermediary group between GCB and ABC clusters originated from non-AIDS donors (181). Taken together, the heterogeneous results on the cellular origin of DLBCL in the HIV+ context indicate that more studies are necessary to elucidate whether there is a subtype prevalence among HIV-DLBCL subjects or if the frequencies vary according to the study population.

Differential miRNA expression was also associated with HIV-DLBCL phenotype. When compared to non-neoplastic B cells from healthy donors, overexpression of miR-17, miR-106a, miR-106b, miR-18a and miR-19a was detected in lymphomas arising in the HIV+ context, including DLBCL. Among those, miR-106a and miR-106b significantly blocked the p21 cell cycle inhibitor and, consequently, enhanced cellular proliferation (182), which corroborates with the increased proliferative behavior already described in HIV-DLBCL. Also, miR-21 expression was higher in HIV-DLBCL than in IC-DLBCL and those patients considered “miR-21 high” exhibited poorer survival (183). Another report demonstrated elevated serum levels of miR-222 in PLWH prior to DLBCL development, suggesting its measurement as a valuable marker for the identification of HIV+ subjects at risk of developing lymphoma before diagnosis (184).

Besides miRNA expression signatures, different chromosomal alterations were reported comparing HIV-DLBCL and IC-DLBCL. A study showed that, although the overall number of genomic alterations was similar between groups, the distribution of certain lesions was significantly associated with HIV status (185). The most common alterations in HIV-DLBCL were deletions in 3p14.3 and gains of 12q21.31, while gains of 18q (a region containing BCL2) were the most frequent rearrangements in IC-DLBCL. The authors also showed that the alterations had functional impact. The chromosomal deletions enriched in HIV+ samples were associated with reduced expression of known tumor suppressor genes, such as WWOX, FHIT, DCC and PARK2 (185), another example of a carcinogenic pathway exclusively detected in HIV-DLBCL. Morton and coworkers (2014) also showed that, among the three most common translocation targets in DLBCL, translocation of MYC was the most frequent in HIV-infected individuals, while translocations of BCL2 or BCL6 were rare (186). In agreement with both previous reports, enrichment of MYC targets, as well as losses affecting WWOX and FHIT were observed in HIV-DLBCL by Deffenbacher and colleagues (2010) (187). Altogether, multiple data show various molecular mechanisms altered specifically in PLWH and support the assumption that HIV-DLBCL is a particular disease with a considerable amount of differences from its manifestation in immunocompetent hosts.

Lastly, few studies have also indicated deregulation of immunological pathways in HIV-related lymphomas (155). For example, the pattern of immunoglobulin gene rearrangement products observed in HIV-DLBCL is particular and differs from that of HIV-uninfected individuals (189). Capello and coworkers (2008) showed an enrichment of IGHV4 family (especially the IGHV4-34 gene) and an underrepresentation of IGHV3 family (in particular, the IGHV3-23 gene) among HIV-DLBCL patients (188). Moreover, serum levels of the soluble forms of CD23 and CD27 were increased in AIDS patients who went on to develop lymphomas, including DLBCL, when compared to HIV+ or even to AIDS subjects who did not progress to cancer (158, 189). Although there is a lack of data addressing immune-related gene expression signatures specifically in HIV-DLBCL, those seminal reports provided valuable insights about immune pathways possibly altered. Interestingly, in immunocompetent subjects, sCD27 was not associated with increased risk of DLBCL (197), corroborating its relevance and biomarker potential in HIV-DLBCL only.

## HIV-Related DLBCL: Particular Clinical Features and Treatment Outcomes

**Figure 3:**
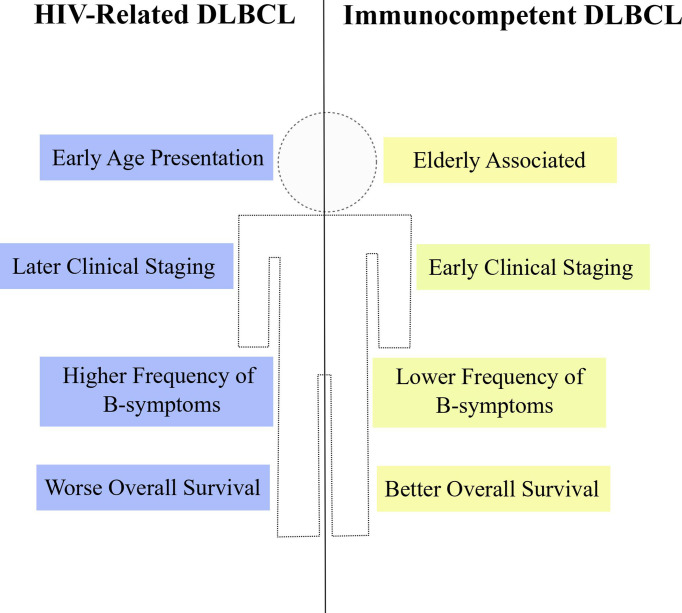
Clinical properties of HIV-DLBCL. HIV-related DLBCL (blue) is commonly associated with particular clinical features when compared to immunocompetent DLBCL (yellow), such as early age at diagnosis, later clinical staging, higher frequency of B-symptoms and worse overall survival estimates.

The HIV+ population not only harbors greater incidence estimates of non-Hodgkin lymphomas (24–26), but also DLBCL is clinically distinct between PLWH and the general HIV-uninfected population. Some reports comparing clinical variables between HIV+ and HIV- people affected by DLBCL or NHLs in general have indicated differences regarding age at diagnosis, tumor staging, frequency of symptoms and frequency of extranodal site involvement (**Figure 3**). A report from Spanish patients with DLBCL described that HIV+ individuals were significantly younger at the time of diagnosis compared to the HIV-uninfected counterparts (median 44 years vs. 62 years, respectively). Also, HIV+ patients with DLBCL exhibited more frequently B-symptoms, later clinical staging (III-IV) and worse ECOG score (≥ 2) than HIV- subjects (198). The younger age at diagnosis appears as a common feature of lymphomas from PLWH and was also noticed in a report studying HIV-related NHL cases in Italy (199). In agreement with those previous reports, data from USA and Puerto Rico confirmed the earlier age presentation, higher frequency of B-symptoms and more advanced clinical staging (III-IV) in HIV+ patients with either DLBCL or NHLs (24, 200). Altogether, the results consistently show particular clinical features of DLBCL in PLWH from different populations worldwide. Additionally, other clinical specificities were reported in the literature. Increased risk of DLBCL occurring at a extranodal site was observed in PLWH when compared to HIV-uninfected patients (25, 200, 201), although this difference was not observed in the work by Baptista and collaborators (198) possibly due to differences in study population and sample size. Among extranodal sites, gastrointestinal tract was significantly more common in HIV+ patients (25, 201, 202) and an increase in central nervous system involvement was also reported (24). It is noteworthy that, in general, the reports did not observe statistical differences between HIV+ and HIV- subjects regarding the international prognostic index (IPI) (25, 198, 203). However, an exception is the work by Spina and collaborators which found worse IPI scores (≥ 2), significantly more frequent in PLWH (201). Taken together, data from different reports indicate that, in fact, the DLBCL (and the NHL as a whole) from PLWH has a particular clinical presentation suggestive of more aggressive features at diagnosis.

Besides analyzing clinical features, some reports have also compared survival estimates between HIV+ and HIV- patients with DLBCL or NHL. The overall survival (OS) of HIV+ subjects with DLBCL was lower than in the HIV-uninfected counterparts even in multivariate models adjusted for type of treatment (24, 198–200). However, differences in the survival estimates according to the HIV status are not clear when analyzing specifically the death by lymphoma instead of death by any cause (overall survival). Indeed, the disease-free survival (DFS) did not differ between HIV-DLBCL and immunocompetent DLBCL (IC-DLBCL) treated with R-CHOP (198). Also, the 2-year lymphoma specific mortality remained unchanged between NHL patients with or without HIV when the former group had CD4 counts of at least 200 cells/μL and no history of other AIDS-defining illnesses, although the ones with less than 200 cells/μL and/or AIDS-defining illness performed worse when compared to non-HIV subjects (24). An analysis with HIV+ patients with different types of cancers showed that there were no differences in cancer-specific mortality for DLBCL subjects stratified according to the HIV status and, in fact, 64.8% of all deaths from PLWH with DLBCL were attributable to HIV-related complications and not to the lymphoma itself (204). Nevertheless, in contrast with those previous reports, Coutinho and collaborators reported unexpected better estimates of both OS and DFS in HIV+ individuals with DLBCL (25). Taken together, the results indicate that the HIV-DLBCL is accompanied by worse OS and equal estimates of lymphoma-specific death, suggesting that, independently of HIV, the response to lymphoma treatment could be similar in both groups. In agreement with that, a systematic review showed that the R-CHOP regimen is a lymphoma treatment associated with improvement in OS and progression-free survival (PFS) in HIV-infected patients. The authors also demonstrate that the use of R-CHOP together with HAART did not affect survival or response to treatment (26), emphasizing the effectiveness of this chemotherapeutic regimen in PLWH. Moreover, the administration of R-CHOP significantly improved the survival and reduced the frequency of death due to lymphoma in HIV-DLBCL patients under HAART treatment (205), which may explain the unchanged estimates of lymphoma-specific mortality seen in previous studies.

In contrast with the data described above (26, 205), the first studies to address the response to chemotherapy in HIV-related DLBCL cases raised the concern of treatment-related toxicity being increased in this group. For example, the work by Kaplan and collaborators indicated that the addition of rituximab to the CHOP drug combination (the R-CHOP regimen) was associated with greater incidence of adverse effects in PLWH (206). The addition of rituximab to the CDE (cyclophosphamide, doxorubicin and etoposide) regimen was also suggestive of increased frequency of adverse events in HIV-related NHL subjects (207). Nevertheless, after these first publications, the feasibility of adding rituximab in different drug combinations for lymphoma treatment in PLWH has been reassessed by several clinical trials. Indeed, different reports showed the effectiveness and safety of rituximab-containing chemotherapeutic regimens for the HIV+ population (26, 208–211) and, interestingly, even in severely immunosuppressed HIV-infected patients (with CD4 counts less than 100 cells/μL), rituximab was associated with improved survival without increasing the rate of adverse effects. A possible difference between reports, as suggested by Dunleavy & Wilson (212), was that HIV+ patients with very low CD4 counts (less than 50 cells/mm3) were the most affected by the treatment-related adverse effects reported primarily by Kaplan and collaborators, while other reports excluded patients with clinical signs of advanced HIV disease (209). In fact, HAART improved the survival in patients with HIV-related B-cell lymphomas (205, 213), as well as low CD4 counts were associated with worse OS in HIV-DLBCL (214). Therefore, despite having more aggressive clinical features at diagnosis, HIV-infected patients are consistently being reported as equally eligible for chemotherapeutic regimens as well as their HIV-uninfected parallels, especially in the context of the HAART era and with comparable CD4 levels.

## Concluding Remarks

HIV-DLBCL is a particular illness with specific characteristics. As shown previously by diverse data, DLBCL from PLWH is accompanied by specific clinical features, such as early age at diagnosis, higher frequency of B symptoms and extranodal involvement, as well as later tumor staging. Interestingly, unique molecular properties are also observed in HIV-DLBCL subjects, including gene expression signatures, chromosomal rearrangements and miRNAs altered levels. All in all, HIV-DLBCL-related properties may occur due to viral modulation of B cell compartments and direct influence during lymphomagenesis. Even though HIV-DLBCL patients consistently exhibit equal estimates of survival when compared to IC-DLBCL subjects (198, 204), PLWH are commonly excluded from clinical trials. In fact, regarding lymphoma-associated clinical trials, the estimates of PLWH exclusion are around 70% (215, 216). Given the intrinsic relationship between lymphoma and HIV, the inclusion of HIV-DLBCL in clinical trial protocols may benefit and improve the understanding of the disease in this particularly susceptible population.

## Author Contributions

PC: wrote the manuscript. PC, FL, and MS: reviewed and edited the manuscript. All authors contributed to the article and approved the submitted version.

## Funding

MS was supported by grants by the Rio de Janeiro State Science Foundation (FAPERJ) # E-26/202.894/2017 and by the Brazilian Research Council (CNPq) # 305765/2015-9. PC is recipient of a Master’s scholarship by the Brazilian National Cancer Institute (INCA).

## Conflict of Interest

The authors declare that the research was conducted in the absence of any commercial or financial relationships that could be construed as a potential conflict of interest.
